# Platelet-Rich Plasma with Endothelial Progenitor Cells Accelerates Diabetic Wound Healing in Rats by Upregulating the Notch1 Signaling Pathway

**DOI:** 10.1155/2019/5920676

**Published:** 2019-08-28

**Authors:** Cheng Zhang, Yu Zhu, Shengdi Lu, Wanrun Zhong, Yanmao Wang, Yimin Chai

**Affiliations:** Department of Orthopedic Surgery, Shanghai Jiao Tong University Affiliated Sixth People's Hospital, Yishan Rd. 600, Shanghai 200233, China

## Abstract

Diabetic wounds, as a kind of refractory wound, are very difficult to heal. Both endothelial progenitor cell (EPC) transplantation and platelet-rich plasma (PRP) can improve diabetic wound healing to some extent. However, PRP application cannot provide reparative cells, while EPC transplantation cannot replenish the required growth factors for wound healing. Thus, when applied alone, neither of these factors is sufficient for effective wound healing. Furthermore, the proliferation, differentiation, and fate of the transplanted EPCs are not well known. Therefore, in this study, we examined the efficacy of combined PRP application with EPC transplantation in diabetic wound healing. Our results indicated that PRP application improved EPC proliferation and migration. The Notch signaling pathway plays a key role in the regulation of the proliferation and differentiation of stem cells and angiogenesis in wound healing. The application of PRP upregulated the Notch pathway-related gene and protein expression in EPCs. Furthermore, experiments with shNotch1-transfected EPCs indicated that PRP enhanced the function of EPCs by upregulating the Notch1 signaling pathway. In vivo studies further indicated that the combination of PRP and EPC transplantation increased neovascularization, reduced wound size, and improved healing in rat wound models. Thus, PRP application can provide the necessary growth factors for wound healing, while EPC transplantation offers the required cells, indicating that the combination of both is a potent novel approach for treating diabetic wounds.

## 1. Introduction

Diabetes is currently the third most serious chronic disease threatening human health, and nearly 415 million people suffer from it globally [1, 2]. Approximately 2% of diabetic patients develop foot ulceration every year, with 14-24% of them requiring amputation [3]. Based on current evidence, the principles of management of diabetic foot ulcers include glycemic control, vascularization improvement, debridement, total contact castings, and offloading as well as appropriate wound dressing [2]. However, a number of patients still remain unhealed after these treatments [4]. In fact, the normal healing pattern is disturbed in diabetic wounds, in which there is a lack of growth factors, excessive inflammation, and a shortage of normal reparative cells. Therefore, diabetic wound healing has become an urgent challenge.

The application of platelet-rich plasma (PRP) is one approach that researchers have been considering to treat diabetic wounds [5]. PRP is packed with various cytokines and proteins essential to wound healing [6] and has been proven to be effective in several animal and clinical trials [7–9]. However, the disorderly release of cytokines may not work out effectively, as wound healing is complicated and relies on functional cell activities and specific cytokine behavior. Endothelial progenitor cells (EPCs) are involved in wound repair and are known to show decreased function and increased apoptosis in diabetic wounds [10]. In a normal wound, tissue ischemia can induce bone marrow-derived progenitor cell mobilization through several pathways and thereby achieve wound repair. However, in diabetic wounds, these conduction pathways are largely dysfunctional. We hypothesized that EPCs, as a functional cell, might conduct the cytokines in PRP for a better outcome.

It is universally known that the Notch signaling pathway is an evolutionarily conserved signaling pathway that affects the normal morphogenesis of fine cells in many developmental processes, including the differentiation of pluripotent progenitor cells, apoptosis, cell proliferation, and the formation of cell boundaries. Recently, many studies have shown that the Notch signaling pathway plays an important role in wound healing, and the activation of this pathway can accelerate wound healing [11]. Thus, in this study, we hypothesized that EPCs in PRP might show better migration and proliferation, and that PRP combined with EPCs might accelerate diabetic wound healing via the activation of the Notch1 signaling pathway.

## 2. Materials and Methods

### 2.1. Extraction and Appraisal of EPCs and Preparation of PRP

EPCs were cultured as described previously [12] and identified by fluorescence staining. PRP was prepared as described previously [13]. The whole blood and PRP were taken for platelet counting to ensure that the number of platelets in PRP was more than four times that in the whole blood. After measuring the platelet count with a whole blood cell analyzer, heparin sodium was added as an anticoagulant at a final concentration of 2 U/mL. PRP was then divided into cryopreservation tubes and frozen and thawed repeatedly for three times using liquid nitrogen. Activated platelet plasma was centrifuged at 3500 g for 25 min, and the supernatant was filtered for subsequent experiments.

### 2.2. Cell Migration Assay

The Transwell system was used to study the effect of PRP on the migration ability of EPCs. EPCs were cultured in medium containing 1% serum 12 h before the experiment. The surface of the upper compartment of the membrane in the Transwell chamber was coated with 50 mg/L Matrigel 1 : 8 diluent solution and dried at 4°C. The residual liquid in the culture plate was sucked out, and then 50 *μ*L serum-free medium containing 10 g/L BSA was added to each well at 37°C for 30 min. EPCs were digested by trypsin to form a cell suspension, washed twice with PBS, and resuspended with serum-free culture medium containing BSA. The cell density was adjusted to 1 × 10^5^ /mL, and then 100-200 *μ*L of the cell suspension was added to the Transwell chamber. Next, 500 *μ*L of medium containing various concentrations of PRP was added to the subchamber of the 24-well plate. Experiment groups include 5%, 10%, and 20% PRP in EGM-MV media. Normal medium was used as the control group. After 24 h of routine culture, the cells were stained and counted under an inverted microscope. The results were compared with those in the conventional culture medium group.

### 2.3. Cell Apoptosis Assay

The cells were incubated with different concentrations of PRP, and then the culture medium was sucked out and placed into a centrifuge tube. Next, 0.2% trypsin was added to the culture dish for digestion, and the cell suspension was formed after blowing. The cell suspension was removed into a centrifuge tube containing culture medium and washed with cold PBS after centrifugation. An Annexin V/PI apoptosis kit (Invitrogen, Carlsbad, CA, USA) was used to detect the percentage of apoptotic cells according to the manufacturer's instructions. After treatment, the cells were detected by flow cytometry.

### 2.4. Cell Proliferation Assay

The cells were incubated with different concentrations of PRP. After adding 20 *μ*L of 5 g/L MTT solution, the cells were cultured for 4 h, and then the supernatant in the culture hole was sucked out. After 10 min of horizontal oscillation, the proliferative activity of the cells in each group was determined by measuring the absorbance at 492 nm in the presence of 150 *μ*L dimethyl sulfoxide (DMSO) per hole.

### 2.5. Establishment of shNotch1 EPCs

GenBank was used to search for the information on rat Notch1, design the Notch1 target, compare the homology after BLAST, and synthesize a double-stranded DNA oligonucleotide. The linearized RNA interference vector pAJ-U6-shRNA-CMV-eGFP/PuroR was ligated with the synthesized double-stranded DNA oligo and transformed into competent cells. Positive clones were selected for identification. To determine transfection efficiency, 293T cells were cotransfected with a three-plasmid lentivirus system, packed, purified, and transfected into EPCs. The expression changes in the Notch1 gene and protein were detected by polymerase chain reaction (PCR) and western blotting, respectively. Observations indicated that the plasmid targeting Notch1 RNAi was constructed successfully, and that lentiviral packaging occurred.

### 2.6. Real-Time PCR (RT-PCR)

The RT-PCR technique was used to detect the expression of the EPC vasoactive factor and the main target gene Hes1 in the Notch1 pathway after PRP treatment. After EPCs were treated with medium containing PRP for 2 days, 1 mL Trizol reagent (Invitrogen) was added to each 10 cm dish and mixed evenly. The total RNA was extracted according to the manufacturer's instruction. Next, 1 *μ*g of the RNA samples was reverse transcribed with oligo dt15 to cDNA. Power SYBR Green PCR Master Mix reagent (Invitrogen) was used to detect the expression of cytokines such as VEGF, PDGF, eNOS, SDF-1, Hes1, Notch1, Jagged-1, Jagged-2, DII-4, Bax, Bcl2, and Caspase3. The forward and reverse primers are listed in Table 1.

### 2.7. Western Blotting

The expression levels of Notch-1, Jagged-1, Jagged-2, and DII-4 proteins were detected by western blotting. After the incubation of EPCs in medium containing different concentrations of PRP for 0, 2, 4, and 6 days, PBS was used to wash the petri dish once, followed by the addition of the protease inhibitor for cell lysis. The protein levels were quantified using a bicinchoninic acid kit (Servicebio Technology, Boston, MA, USA). After polyacrylamide gel electrophoresis, equal amounts of proteins were transferred to the PVDF membrane. The samples were then treated with 5% skimmed milk powder for 1 h at about 25°C and then incubated overnight with 1 : 1000 primary antibody (Sigma-Aldrich, St. Louis, MO, USA) at 4°C, washed with TBST thrice, and incubated with horseradish peroxidase-conjugated secondary antibodies at room temperature for 1 h. The samples were washed thrice with TBST and then detected using the chemiluminescence method. The results were determined relative to the optical density of GAPDH.

### 2.8. Diabetic Wound (DM) Rats

Nonobese rats with diabetes mellitus/severe combined immunodeficiency were injected intraperitoneally with 50 mg/kg of STZ (Sigma-Aldrich) after fasting for 12 h, and rats in the normal group received citrate buffer solution intraperitoneally as control. On the 3rd, 5th, and 7th day after injection, the blood glucose level of the rats was determined. The blood glucose levels of the diabetic model rats at 3, 5, and 7 days after injection were all higher than 16.7 mmol/L.

After the diabetic model was successfully established and the blood glucose was stabilized above 16.7 mmol/L, ketamine was injected intraperitoneally. The rats were placed in a prone position, and their backs were shaved. A 2 × 2 cm^2^ square wound was formed by cutting off the whole layer of skin between the bilateral scapula and retaining the subdermal membrane.

### 2.9. Study Design

The diabetic wound model was established in SD rats, which were then divided into five groups: the control group (normal saline), the EPC group, the PRP group, the 10% PRP+shNotch EPC group, and the 10% PRP + EPC group. Each group included eight rats. Different concentrations of PRP-EPC suspension were used to cover around and the bottom of the wound, while the control group was treated locally with normal saline. After routine feeding, the wound condition was examined every other day, and the wound area, epithelization, and wound healing were observed. The wound tissue was taken 4, 7, 10, and 14 days after the operation for wound examination, and samples of the 14th day were subjected to histological examination.

### 2.10. Hematoxylin and Eosin (H&E), Masson, CD31, and *α*-SMA Staining

For histological examination, the wound tissue was fixed with paraformaldehyde and embedded in paraffin and then stained with H&E, and the proliferation of the wound cells was detected by an optical microscope. Collagen synthesis was detected by Masson staining as described below. Tissue paraffin slices were dewaxed by gradient alcohol to water and then stained with hematoxylin staining solution for 5-10 min. Next, the samples were rinsed with 2% glacial acetic acid solution for a while, followed by the addition of 1% phosphomolybdic acid aqueous solution for 3-5 min, and then aniline blue or light green solution for 5 min. The samples were washed with Masson Richun red acid complex red solution for 5 min and then washed with 2% glacial ice acetic acid solution for a while. Then, the samples were taken out and soaked in 0.2% acetic acid solution and sealed in 95% alcohol, anhydrous alcohol, xylene transparent, and neutral tree gum. The sections were examined under an optical microscope, and the collagen synthesis of each group was compared. Angiogenesis was detected by CD31 immunohistochemical staining and *α*-SMA staining, which marks the mature blood vessel. The specimens were fixed in 10% formalin solution, buried in paraffin, and cut into sections of 5 *μ*m thickness. After dewaxing with water and gradient alcohol, sodium citrate was added to repair antigens. The samples were then labeled with an anti-CD31 polyclonal antibody (Biotech, USA) and stained with DAB and examined under an optical microscope.

### 2.11. Statistical Analysis

SPSS 17.0 software was used for statistical analyses. All data are expressed as mean ± SEM. Statistical analysis was carried out using paired 2-tailed Student's *t*-test and ANOVA. *p* < 0.05 was considered statistically significant. ImageJ was used to analyze the images.

## 3. Results

### 3.1. PRP Promoted the Proliferation and Immigration of EPCs, Especially in 10% PRP

**Figure 1:**
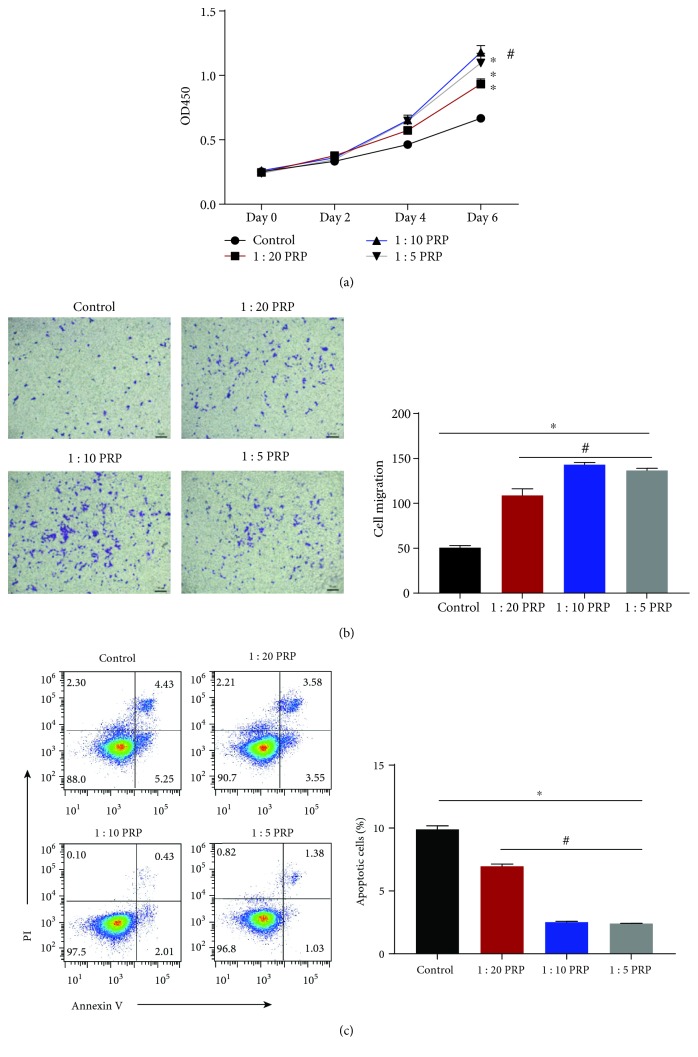
EPC apoptosis, proliferation, and migration in each group. (a) EPC proliferation in each group at days 0, 2, 4, and 6 is shown as the OD450 value. (b) EPC migration assay. The number of migrating EPCs was analyzed and is shown as a bar chart. (c) The percentage of apoptotic EPCs is shown as a bar chart. ^∗^*p* < 0.05 for the treatment group vs. the control group and ^#^*p* < 0.05 for the 1 : 10 PRP group vs. the other concentration groups.

The proliferation and migration ability of EPCs in PRP was evaluated by using cell migration and proliferation assays. Compared with the respective control groups, EPCs showed better proliferation and immigration in the PRP-treated groups (Figures 1(a) and 1(b)); the OD450 value in the 10% PRP-treated group was one-fold higher than that in the control group, and the number of migrated EPCs was nearly three-fold compared to that in the control group. Particularly, 10% PRP seemed to be the optimal concentration for EPCs among the three different concentrations used. In addition, flow cytometry was used to determine the apoptosis of EPCs (Figure 1(c)). EPCs showed lower rates of apoptosis in PRP-treated groups than in control groups, while the 10% PRP groups showed the least apoptosis rate among these groups.

### 3.2. Notch1 Pathway-Related and Angiogenic Genes and Proteins Showed Increasing Trends in PRP-Treated EPCs

**Figure 2:**
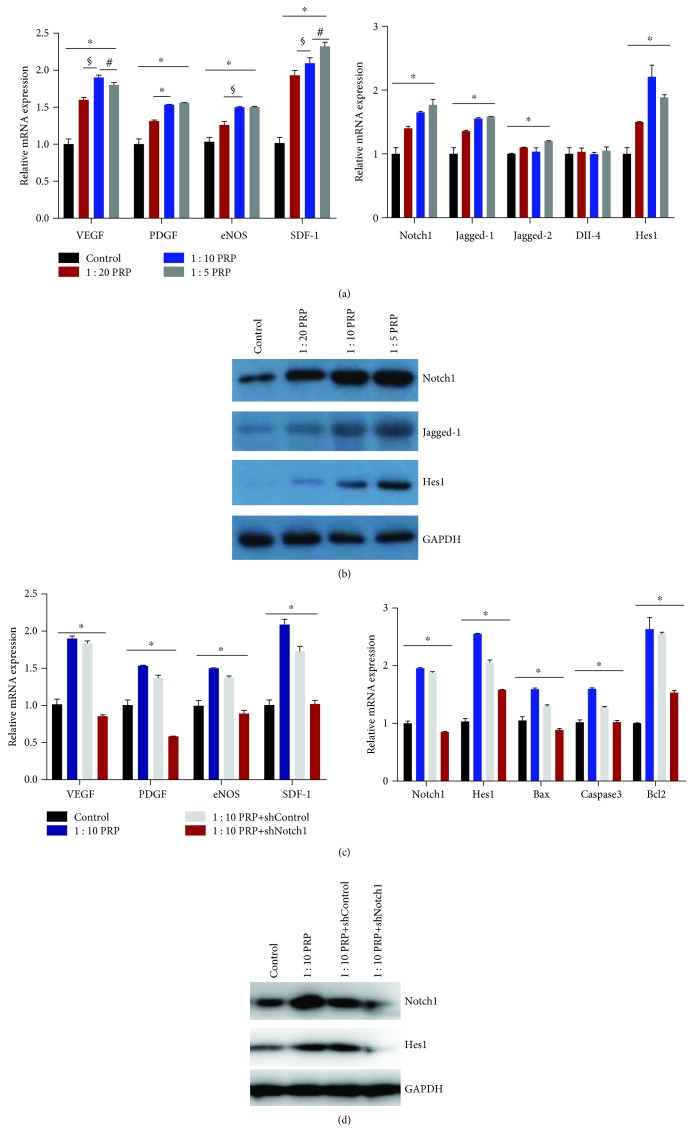
qPCR and western blotting results of angiogenic and Notch 1 pathway-related genes in EPC. There were significant differences between the control and treatment groups. In addition, the 1 : 10 PRP groups had several genes that were significantly upregulated compared with those in the other groups. (a and c) Gene expression changes by qPCR. (b and d) Protein expression levels by western blotting. ^∗^*p* < 0.05 for the treatment groups vs. the control group and for the shNotch group vs. the other groups, ^§^*p* < 0.05 for the 1 : 10 PRP group vs. the 1 : 20 PRP group, and ^#^*p* < 0.05 for the 1 : 10 PRP group vs. the 1 : 5 PRP group.

Western blotting and qPCR were used to assess the expression of Notch1 pathway-related proteins and genes in EPCs. In our experiment with EPCs, Notch 1 and its downstream proteins including Jagged-1 and Hes1 showed significantly higher expression levels than those in the control group (Figure 2(a)). The expression of proteins was upregulated in the PRP-treated groups compared to that in the control group (Figure 2(b)).

### 3.3. shNotch1 EPCs Did Not Show Upregulated Expression of Angiogenic and Notch1 Pathway-Related Genes and Proteins

shNotch1 EPCs were established through low-virus transfection. Western blotting and qPCR were used to evaluate the changes in gene expression in PRP-shNotch1 groups. In the EPCs, angiogenic genes such as VEGF, PDGF, eNOS, and SDF-1 were significantly upregulated in PRP-treated groups compared with those in control groups (Figure 2(c)). Nevertheless, shNotch1 EPCs showed no upregulation of these genes when compared to the 10% PRP-treated and control groups (Figure 2(c)). In addition, the expression of Notch 1 and its downstream genes such as Notch 1, Jagged-1, Jagged-2, DII-4, and Hes 1 was also found to be increased, and the 10% PRP group showed higher expression levels. In the shNotch1 EPC group, there were no significant changes in the expression of Notch1 pathway-related genes when compared to the control group (Figure 2(c)). Western blotting results showed a similar trend (Figure 2(d)).

### 3.4. PRP Affected EPC Function, including Proliferation and Migration, by Upregulating Angiogenic and Notch 1 Pathway-Related Genes

**Figure 3:**
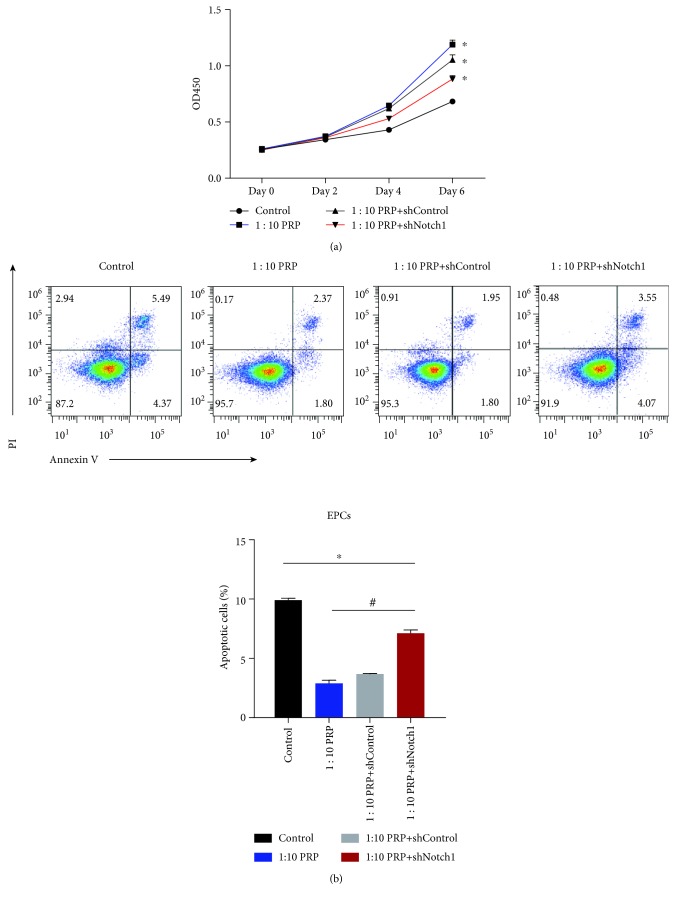
(a) Proliferation of the EPCs shown by the OD450 values at days 0, 2, 4, and 6. (b) Percentage of apoptotic EPCs shown as a bar chart. ^∗^*p* < 0.05 for the treatment groups vs. the control group and ^#^*p* < 0.05 for the 1 : 10 PRP + shNotch1 group vs. the other treatment groups.

Cell proliferation and apoptosis assays were carried out in shNotch1 EPCs and other groups. The shNotch1 EPC group wherein the normal Notch1 pathway was disrupted showed lower proliferation rates and higher apoptosis rates than the PRP and PRP-ShControl EPC groups, although the proliferation rates in this group were better than those observed in the untreated control group (Figure 3).

### 3.5. In Vivo, PRP Associated with EPCs Reduced Wound Sizes and Promoted Healing in a Rat Model of Diabetic Wound

**Figure 4:**
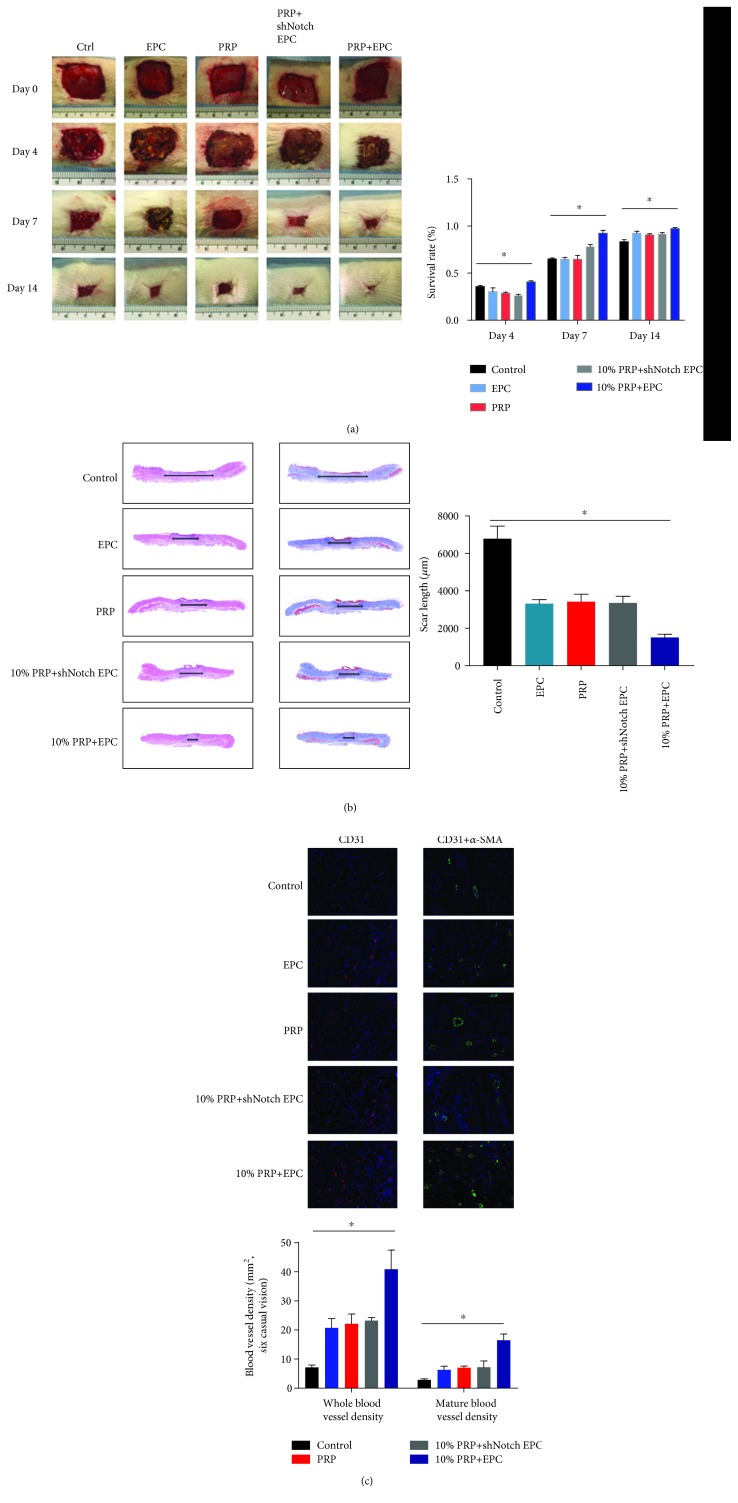
In vivo test. (a) Wound situation and wound size, immediately after operation and at days 4, 7, and 14. Treatment groups showed a significant difference compared with the control group, ^∗^*p* < 0.05. (b) H&E and Masson staining of specimens at day 14. (c) CD31 and *α*-SMA immunostained samples of rats at day 14. ^∗^*p* < 0.05 for the treatment groups vs. the control group.

In DM rats, 2 cm × 2 cm wounds were made. PRP-EPC groups showed the smallest wounds at day 14 when compared with those in the other groups; in general, control groups showed poor outcomes (Figure 4(a)). In addition, the H&E and Masson staining methods were used to evaluate wound healing and collagen regeneration. We found that PRP-EPC groups gave better results than the other groups, and PRP-shNotch1 EPC groups did not show significant differences when compared to the PRP or EPC groups (Figure 4(a)). Likewise, CD31 staining showed similar results, where PRP-EPCs showed a significantly higher number of mature and total blood vessels than the other groups (Figure 4(a)).

### 3.6. PRP Associated with EPCs Resulted in Increased Neovascularization and Promoted Healing

H&E and Masson staining were used to assess collagen formation and wound healing. The associated treatment group showed a significantly smaller scar length than the other groups (Figure 4(b)). In addition, CD31 immunohistochemical staining and *α*-SMA staining were used to evaluate blood vessel regeneration. The 10% PRP-EPC groups showed more CD31+ staining than the other groups. CD31+*α*-SMA staining showed the same trend (Figure 4(c)).

## 4. Discussion

Diabetic wounds are refractory owing to various factors, including the lack of normal reparative cells and stimulation of several cytokines [14]. Considering the large number of patients with diabetic wounds, effective treatment methods should be explored. However, current treatments used by many specialists have shown limited success, and hence a novel method for treating diabetic wounds should be developed.

In view of the lack of normal reparative cells, EPCs are gradually attracting attention. EPCs can be recruited to ischemic areas to repair blood vessels by transdifferentiating into vascular endothelial cells [15]. It is reported that the Notch1 and PDGF signaling pathways participate in vascular repair and neovascularization regeneration in wound healing [16, 17]. The Notch1 signaling pathway is highly conserved, which is responsible for intercellular transduction and differentiation [18]. Li et al. [19] reported that EPCs, which can repair damaged blood vessels and have angiogenetic abilities, are possibly regulated through the activation of the Notch signaling pathway. Other scholars also tried to explore the mechanism underlying the action of EPCs and wound healing, including the activation of Erk1/2 signaling [20]. However, in diabetic wounds, high levels of reactive oxygen species are produced, inducing an EPC deficit. Their abilities of angiogenesis, proliferation, differentiation, migration, and adhesion are impaired [21]. Therefore, many researchers have investigated EPCs for wound management in experimental studies. Suh et al. [22] reported that in a mouse wound, EPC-treated groups exhibited a better wound-closure rate due to recruiting monocytes/macrophages and improving neovascularization. Recently, it was reported that EPCs could accelerate wound healing in a diabetic swine model [23]. However, cell therapy alone can result in up to 20% of wounds remaining unhealed [24]. Researchers believe that this lack of efficacy is possibly due to the lack of several cytokines that promote the migration and survival of EPCs. Wounds are an inflammatory environment in which growth factors are aberrant, so even if exogenous EPCs are injected, their function will be impaired owing to the lack of stimulation by growth factor stimuli. Hence, EPCs alone are insufficient for treatment of diabetic wounds.

Interestingly, with the increasing application of PRP, which is packed with several growth factors, many experts attempted to add PRP onto diabetic wounds, which was found to be beneficial for wound healing and achieved better prognosis [25–28]. In a pioneering study, 32 patients with chronic, nonhealing, cutaneous wounds of the lower extremities achieved quite satisfactory effects after treatment with a PRP formula [29]. PRP can alleviate inflammation and control local blood sugar, which is favorable for cell proliferation, homing, and migration, thereby accelerating wound healing. More recently, Li et al. [30] reported that 84.8% of patients in a clinical trial achieved healing after treatment with an autologous platelet-rich gel. However, in this trial, 15.2% of the patients remained unhealed with a risk of amputation.

Considering the several growth factors present in PRP, EPCs could be positively affected by them [30]. Thus, we started to focus on combining PRP with EPCs as a novel therapy. In our study, we suggested that PRP, which is rich in many factors, can regulate EPCs effectively in order to promote wound healing via upregulating the classical Notch1 signaling pathway. In vitro, the cell migration, apoptosis, and migration assays showed that PRP could largely improve EPC migration and proliferation; the best concentration of PRP for EPCs is suggested to be 10%. When the EPC Notch1 signal was disrupted, PRP exerted no positive effect. Furthermore, we found that angiogenic and Notch1 signal-related genes were upregulated in PRP-treated EPCs, which attested that PRP could regulate the functions of EPCs via the Notch1 signaling pathway. In vivo, this therapy promoted healing in diabetic wound rats and highly upregulated angiogenic and Notch1 pathway-related genes. We found that DM rats subjected to this therapy exhibited better healing outcomes. Therefore, it is suggested that PRP combined with EPCs may be a potential therapeutic option for diabetic wounds.

## 5. Conclusions

In conclusion, diabetic wounds are refractory and difficult to heal. PRP, as a rising weapon, could activate EPCs by upregulating the Notch1 signaling pathway, thus favoring the healing of diabetic wounds. PRP and EPCs are easy to obtain, and hence have promising prospects for clinical applications. Nevertheless, we believe that PRP, which is a complex of many growth factors and platelets, may exert positive effects on EPCs via several mechanisms but not by the upregulation of the Notch1 signaling pathway. In the future, the precise mechanism underlying the changes in diabetic wounds after this therapy should be explored.

## Figures and Tables

**Table 1:** 

Gene	Sequences	PCR product (bp)
VEGF	5′-GGAGAGATGAGCTTCCTGCAGC-3′	336
	5′-CCTTGGCTTGTCACATCTGCAA-3′	

PDGF	5′-GTAGGACTGCTCAGTTCAAACAT-3′	444
	5′-ACAGTTACTACACCCGTAAGGC-3′	

eNOS	5′-GTACCGGCTGAGTACCCAAGCT-3′	360
	5′-TCCCTCCTGGCTTCCAGTGT-3′	

SDF-1	5′-TGAGAGCCATGTCGCCAGAG-3′	511
	5′-TCACACCTCTCACATCTTGAGCCT-3′	

Hes1	5′-GCTAAGGTGTTTGGAGGCT-3′	122
	5′-CCGCTGTTGCTGGTGTA-3′	

Notch1	5′-CCGCAGTTGTGCTCCTGAA-3′	109
	5′-ACCTTGGCGGTCTCGTAGCT-3′	

Jagged-1	5′-TCGCTGTATCTGTCCACCTG-3′	227
	5′-AGTCACTGGCACGGTTGTAG-3′	

Jagged-2	5′-GGGCTCTTGCCACGAAGT-3′	479
	5′-CATCCACCAGGTCCTCACAG-3′	

DII-4	5′-AAGAATAGCGGCAGTGGTCGTAA-3′	161
	5′-CCTTGGATGATGATTTGGCTGA-3′	

Bax	5′-CCGAGCTGATCAGAACCATCAT-3′	326
	5′-TCTTCCAGATGGTGAGTGAGGC-3′	

Bcl2	5′-GGACAACATCGCTCTGTGGATG-3′	255
	5′-TTGTGGCCCAGGTATGCACC-3′	

Caspase3	5′-GGTTCATCCAGTCGCTTTG-3′	99
	5′-ATTCTGTTGCCACCTTTCG-3′	

GAPDH	5′-GTCTCCTCTGACTTCAACAGCG-3′	131
	5′-ACCACCCTGTTGCTGTAGCCAA-3′	

## Data Availability

The data used to support the findings of this study are available from the corresponding author upon request.
